# Psychometric validation of a multidimensional health facility HIV stigma instrument among healthcare workers in Taiwan

**DOI:** 10.1186/s12889-026-28284-2

**Published:** 2026-06-27

**Authors:** Hsin-Hao Lai, Shan-Lin Liu, Chien Chun Wang, Tsen-Fang Yen, Chih Chieh Tsai, Su-Han Hsu

**Affiliations:** 1 Section of Infectious disease, Taipei City Hospital Yangming Branch, Taipei, Taiwan; 2 School of Medicine, College of Medicine, National Yang Ming Chiao Tung University, Taipei, Taiwan; 3 Section of Infectious Diseases, Chinese Medicine, and Kunming Branch, Taipei City Hospital, Taipei, Linsen Taiwan; 4 Department of Nursing, Chinese Medicine, and Kunming Branch, Taipei City Hospital, Taipei, Linsen Taiwan; 5 Renai Branch, Developing and Planning Department, Taipei City Hospital, Taipei, Taiwan; 6 Department of Health and Welfare, University of Taipei, Taipei, Taiwan; 7 Department of Pharmacy, Taipei City Hospital Yangming Branch, Taipei, Taiwan; 8 School of Pharmacy, College of Pharmacy, Taipei Medical University, Taipei, Taiwan; 9 Department of Physical Education, University of Taipei, Taipei, Taiwan; 10 Department of exercise and health sciences, University of Taipei, Taipei, Taiwan

**Keywords:** HIV stigma, Healthcare workers, Psychometric validation, Structural stigma, WHO stigma tool, Taiwan

## Abstract

**Background:**

HIV-related stigma within healthcare settings remains a major barrier to HIV prevention, treatment engagement, and equitable care. Existing stigma instruments in Taiwan primarily assess individual-level attitudes and do not adequately capture institutional or structural determinants of stigma. A multidimensional health facility–level stigma instrument is needed to support routine monitoring and stigma-reduction efforts. This study aimed to culturally adapt and psychometrically validate a Traditional Chinese version of the WHO/USAID Health Facility Staff HIV Stigma and Discrimination Questionnaire for use among healthcare workers in Taiwan.

**Methods:**

A cross-sectional psychometric study was conducted using three independent samples. Content validity was evaluated by an expert panel. Item analysis was performed in a pretest sample (*n* = 157). The main validation sample (*N* = 619) was randomly divided into Subsample A (*n* = 310) for exploratory factor analysis and Subsample B (*n* = 309) for confirmatory factor analysis. Construct validity, convergent and discriminant validity, and reliability were evaluated. External convergent validity was examined using correlations with the Taiwanese version of the Health Care Provider HIV/AIDS Stigma Scale (TW-HPASS).

**Results:**

Exploratory factor analysis identified a seven-factor structure consisting of Fear, Extra Infection Precautions, Observed Enacted Stigma, Secondary Stigma, Health Facility Policies and Work Environment, Attitudes toward PWH, and Willingness to Treat Key Populations, explaining 72.98% of total variance. Confirmatory factor analysis demonstrated acceptable model fit (CFI = 0.916, TLI = 0.902, RMSEA = 0.069). Composite reliability values ranged from 0.794 to 0.954, and average variance extracted supported convergent validity. Domains conceptually aligned with HPASS showed moderate-to-strong correlations (*r* = .362–0.691), whereas structural domains such as observed stigma and institutional policies demonstrated weak or negligible correlations, indicating that the instrument captures distinct facility-level dimensions of stigma beyond individual attitudes.

**Conclusions:**

The Traditional Chinese WHO/USAID Health Facility Staff HIV Stigma and Discrimination Questionnaire demonstrates strong psychometric properties and provides a multidimensional framework for assessing HIV-related stigma in Taiwanese healthcare settings. By capturing individual, interpersonal, and structural dimensions of stigma, the instrument may support facility-level monitoring, cross-institutional benchmarking, and the development of targeted stigma-reduction interventions.

**Supplementary Information:**

The online version contains supplementary material available at https://doi.org/10.1186/s12889-026-28284-2.

## Background

### Global and healthcare‑setting HIV stigma

HIV-related stigma remains a major barrier to effective HIV prevention and treatment despite substantial biomedical progress. Despite major advances in antiretroviral therapy and global progress toward the UNAIDS 95–95–95 targets [1], HIV-related stigma within healthcare settings continues to undermine multiple stages of the HIV care continuum by discouraging individuals from seeking testing, engaging in care, and achieving viral suppression [2]. A recent pooled analysis conducted by Doyle et al. across multiple African settings demonstrated that people with HIV (PWH) who anticipated or experienced healthcare stigma had up to 14% lower antiretroviral therapy uptake and up to 16% lower viral suppression after accounting for selection bias. The persistence of stigma in clinical environments, where PWH should receive equitable and evidence-based care, reflects discriminatory attitudes, fear-driven behaviors, and institutional practices that compromise care quality and violate human rights. Recent monitoring by the European Centre for Disease Prevention and Control (ECDC) similarly identifies healthcare‑setting stigma as an ongoing challenge and emphasizes the need for standardized tools to track stigma and guide national HIV responses [3]. Together, these findings underscore the need for systematic measurement and targeted intervention to reduce stigma and strengthen HIV care outcomes.

### Multilevel conceptual framework of HIV stigma

Understanding HIV stigma requires a multilevel conceptual framework that explains how stigma is produced and sustained across ecological levels. Contemporary stigma frameworks conceptualize HIV-related stigma as a multilevel and multidimensional process operating across individual, interpersonal, organizational, and structural domains [4–6]. At the individual level, stigma encompasses healthcare workers’ attitudes, fears, and misconceptions that manifest as perceived, anticipated, internalized, or enacted stigma [5]. Perceived stigma refers to individuals’ awareness of negative societal attitudes; anticipated stigma reflects expectations of future discrimination; internalized stigma involves adopting negative beliefs about oneself; and enacted stigma refers to actual experiences of discriminatory behavior. Interpersonal-level stigma involves discriminatory behaviors and social norms within clinical teams, including observed enacted stigma and peer-reinforced practices that influence provider behavior. Structural-level stigma is embedded in institutional policies, organizational procedures, and broader social structures that systematically disadvantage PWH [6, 7]. Recent theoretical work further emphasizes that these levels operate through interconnected pathways that ultimately shape health-related outcomes. Earnshaw et al. [8] describe a multilevel stigma framework in which structural conditions shape interpersonal interactions and social norms, which subsequently influence individual‑level psychological processes such as anticipated, internalized, and perceived stigma. These cascading pathways help explain how stigma becomes embedded within healthcare environments and ultimately affects engagement in care, treatment adherence, and health outcomes. Complementing this perspective, The Health Stigma and Discrimination Framework distinguishes between stigma drivers such as fear of HIV transmission, negative attitudes, and misconceptions, and stigma manifestations including enacted, anticipated, and internalized stigma, which influence outcomes across ecological levels [9]. By integrating these theoretical insights, the WHO/USAID instrument translates individual‑, interpersonal‑, and structural‑level mechanisms into measurable domains, including fear of transmission, observed enacted stigma, and institutional policies. Together, these frameworks underscore that effective stigma reduction requires addressing not only individual healthcare workers’ attitudes but also the organizational and structural conditions that perpetuate discrimination [7, 10].

### Existing stigma instruments and gaps in taiwan

Although several instruments have been developed to measure HIV-related stigma in healthcare settings, the People Living with HIV Stigma Index 2.0 captures experienced stigma from the perspective of PWH but does not assess healthcare workers’ attitudes or facility-level determinants [11]. The Health Care Provider HIV/AIDS Stigma Scale (HPASS) measures individual-level stigma across prejudice, stereotyping, and discrimination, yet does not capture institutional policies, observed stigma behaviors, or attitudes toward key populations [12]. In contrast, the WHO/USAID Health Facility Staff HIV Stigma and Discrimination Questionnaire was designed to assess both individual-level drivers (e.g., fear of transmission, negative attitudes) and facility-level factors (e.g., policies, observed stigma, unnecessary precautions). This tool has demonstrated cross-cultural applicability in diverse settings including China, Dominica, Egypt, Kenya, Puerto Rico and St. Christopher & Nevis [13]. Subsequent adaptations in Thailand, Kazakhstan, and other contexts have further validated its utility while highlighting the importance of cultural adaptation to ensure contextual relevance [14, 15].

In Taiwan, a Traditional Chinese version of HPASS (TW-HPASS) has been developed through systematic cross-cultural adaptation and psychometric evaluation procedures based on the original instrument, providing a culturally relevant framework for assessing HIV-related stigma among healthcare personnel. Preliminary psychometric findings have been presented at a national public health academic conference in Taiwan [16, 17]. However, TW-HPASS does not capture system-level dimensions such as institutional policies, observed stigma within facilities, secondary stigma affecting healthcare workers, or attitudes toward key populations. These limitations restrict the ability to comprehensively assess the multilevel nature of stigma in healthcare settings and to design interventions that address both individual and organizational determinants simultaneously.

### Rationale for adapting the WHO/USAID health facility stigma tool

A culturally adapted health facility–level stigma instrument is needed in Taiwan to capture the full spectrum of stigma across individual, interpersonal, and structural domains. The WHO/USAID Health Facility Staff HIV Stigma and Discrimination Questionnaire offers a more comprehensive framework by integrating individual attitudes, fear-based behaviors, observed stigma, institutional policies, and willingness to serve key populations into a single measurement tool [13]. This multidimensional structure is consistent with contemporary stigma theory, which underscores the necessity of addressing stigma across multiple ecological levels, namely the individual, interpersonal, organizational, and structural environments that shape the production and perpetuation of stigma [7, 9]. Furthermore, the WHO instrument’s focus on programmatically actionable drivers, such as fear of HIV transmission through casual contact and negative attitudes toward PWH, facilitates the design of targeted interventions [7, 10]. By capturing both individual and institutional dimensions, the tool enables healthcare facilities to monitor stigma as part of routine quality‑improvement processes and evaluate the effectiveness of stigma‑reduction programs over time [15, 18, 19]. Given Taiwan’s universal health coverage, centralized HIV care delivery, and mandatory reporting system, these structural features may shape healthcare workers’ perceptions of risk and institutional practices, further underscoring the need for a tool capable of capturing both individual and structural determinants of stigma.

Cross-cultural adaptation is essential to ensure linguistic accuracy and contextual relevance when implementing stigma measurement tools across settings [20]. HIV‑related stigma is shaped by local norms, values, and healthcare system characteristics, and evidence from East Asia indicates that anticipated stigma tends to be higher in this region compared with other global contexts [21]. In Hong Kong, stigma has been closely linked to perceptions of norm violation and moral judgment, reflecting culturally embedded expectations surrounding sexuality and illness [22]. In Taiwan, qualitative studies similarly document that PWH experience stigmatization across multiple settings, including healthcare, particularly related to disclosure [23]. These contextual factors highlight the need for culturally sensitive measurement approaches that reflect how stigma is expressed within Taiwanese clinical environments. In Taiwan, cultural norms surrounding sexuality, disclosure, and moral judgment intersect with system-level features, including universal health coverage, centralized HIV care delivery, and mandatory reporting, to shape healthcare workers’ perceptions of occupational risk, confidentiality, and responsibility. This interplay underscores the importance of using instruments capable of capturing both individual and structural determinants of stigma.

Despite increasing recognition of HIV-related stigma as a barrier to optimal care in Taiwan, no validated healthcare facility-level stigma instrument currently exists that captures both individual attitudes and institutional factors. The present study therefore aimed to culturally adapt and psychometrically validate a Traditional Chinese version of the WHO/USAID Health Facility Staff HIV Stigma and Discrimination Questionnaire for use among healthcare workers in Taiwan. Specifically, this study sought to establish a multidimensional instrument capable of assessing individual‑, interpersonal‑, and structural‑level dimensions of HIV‑related stigma within healthcare settings.

## Methods

### Instrument development

#### Source instrument and cultural adaptation

The present study adopted the Chinese version of the Health Facility Staff Stigma and Discrimination Questionnaire originally developed by Nyblade et al. [13]. under the United States Agency for International Development (USAID) Health Policy Project in 2013. This field-tested instrument was designed to assess HIV-related stigma and discrimination among healthcare workers in clinical settings. The Chinese standardized version released in October 2013 served as the basis for this study [24] .

Although a Chinese version was available, additional contextual refinement was undertaken to ensure linguistic clarity and alignment with the Taiwanese healthcare system. The cultural adaptation process involved forward translation, expert review, and content validation. An expert panel reviewed each item for linguistic accuracy, clarity, cultural appropriateness, and relevance to Taiwanese healthcare settings. Revisions focused on improving terminology consistency, adapting healthcare-related expressions to local clinical practice, and clarifying item wording.

#### Content review

An expert panel consisting of ten members—one head nurse in infectious diseases, two infectious disease chiefs, three questionnaire design specialists, three HIV case managers, and one representative from the national HIV society—each with more than 10 years of relevant professional experience, evaluated the relevance and clarity of all items. These panel members had previously participated in the cultural adaptation and validation of the Taiwanese version of the Health Care Provider HIV/AIDS Stigma Scale (TW-HPASS), providing relevant expertise for the current study.

Content validity was assessed using the Content Validity Index (CVI). Each item was rated on a four-point scale, and item-level CVI values were calculated. Items with I-CVI values below 0.90 were flagged for revision or deletion.

### Psychometric evaluation

#### Study design and samples

Data were collected between September and December 2025 using an anonymous web-based questionnaire. Participants were recruited through hospital-wide announcements and voluntary participation. The study was approved with a waiver of signed informed consent by the institutional review board. An electronic participant information sheet was presented on the first page of the survey, and participants who voluntarily proceeded to complete the questionnaire were considered to have provided informed consent.

Upon completion of the survey, participants received a NT$100 (approximately US$3) convenience-store gift voucher as compensation for their time. To enhance data quality, the online survey platform required completion of all study items before submission. As a result, no missing data were observed in the final dataset. Responses were also reviewed for potential careless responding, including implausible response patterns and excessive missingness; no evidence of invalid response patterns was identified. Therefore, no imputation procedures were required.

This cross-sectional validation study consisted of three independent samples. First, a pretest sample (*n* = 157) was used to conduct item discrimination and preliminary reliability analysis. Second, a main validation sample of 619 healthcare workers was recruited from hospital settings. Participants represented diverse professional roles, including nurses, physicians, pharmacists, medical technologists, therapists, administrative staff, and case managers. The total validation sample was randomly divided into two independent subsamples: Subsample A (*n* = 310) for exploratory factor analysis (EFA) and Subsample B (*n* = 309) for confirmatory factor analysis (CFA). The total sample size met recommended participant-to-item ratios for factor analysis.

#### Item analysis

Item analysis was conducted using the independent pretest sample (*n* = 157). Discrimination was examined using the critical ratio method. Internal consistency was assessed using Cronbach’s alpha. Items demonstrating non-significant discrimination or negatively affecting reliability were considered for removal before factor analysis [25, 26].

#### Construct validity

Construct validity was evaluated using both exploratory factor analysis (EFA) and confirmatory factor analysis (CFA).

EFA was conducted using principal component analysis (PCA) with oblimin rotation in Subsample A. Although PCA is conceptually distinct from common-factor extraction methods and is primarily used as a data-reduction technique, it was selected because the primary objective of the exploratory phase was to examine the dimensional structure of the culturally adapted questionnaire and support item reduction. The resulting factor structure was subsequently evaluated using confirmatory factor analysis in an independent validation sample. Sampling adequacy was evaluated using the Kaiser–Meyer–Olkin measure and Bartlett’s test of sphericity. Items were retained based on primary loadings, absence of problematic cross-loadings, and conceptual interpretability [27].

CFA was conducted using structural equation modeling with AMOS in Subsample B. Model fit was evaluated using multiple indices, including chi-square statistics, χ²/df ratio, Comparative Fit Index, Tucker–Lewis Index, Goodness-of-Fit Index, Root Mean Square Error of Approximation, and Standardized Root Mean Square Residual [28].

Convergent validity was assessed using composite reliability (CR) and average variance extracted (AVE). Discriminant validity was evaluated using the Fornell–Larcker criterion [29].

External convergent validity was examined in the validation subsample (Subsample B, *n* = 309) to avoid overlap with exploratory analyses conducted in Subsample A. Correlations were assessed using the Taiwanese version of the Healthcare Provider HIV/AIDS Stigma Scale (TW-HPASS), a culturally adapted instrument derived from the original HPASS for measuring HIV-related prejudice, stereotyping, and discrimination among healthcare personnel. Conceptually aligned constructs were hypothesized to demonstrate moderate-to-strong positive correlations (*r* ≥ .40), whereas structurally distinct domains (e.g., health facility policies) were expected to show weaker associations. Pearson correlation coefficients were calculated to assess cross-instrument relationships.

#### Reliability

Internal consistency of the final scale and subscales was evaluated using Cronbach’s alpha and composite reliability coefficients.

## Results

### Instrument development

#### Content validity

All items were reviewed by an expert panel to ensure linguistic clarity, cultural appropriateness, and conceptual equivalence with the original instrument. Item-level content validity indices (I-CVI) met the predetermined threshold, indicating satisfactory content validity. Minor wording refinements were made to enhance clarity in the Taiwanese healthcare context. Examples included modifications to infection-control terminology and refinements of clinical care scenarios to improve comprehensibility. No items were removed during the cultural adaptation process.

Although the original instrument employed mixed response formats (e.g., yes/no, frequency-based responses), all items in the present study were standardized to a Likert-type response format to improve measurement consistency and psychometric robustness. The conceptual meaning of each item was preserved during this adaptation process.

### Psychometric evaluation

#### Participant Characteristics

A total of 157 healthcare workers participated in the pretest phase for item analysis and preliminary reliability assessment. The main validation sample consisted of 619 healthcare workers. The mean age was 42.29 years (SD = 11.10). Women comprised 78.2% (n = 485) of the sample, and men comprised 21.6% (n = 134). Regarding education, 58.5% held a bachelor’s degree and 17.9% held a master’s degree. Nurses constituted the largest professional group (39.5%), followed by administrative staff (17.0%) and physicians (7.0%). More than half of participants had over 10 years of clinical experience, and 70.27% reported participation in HIV-related training. The mean number of people living with HIV served in the past month was 11.82 (SD = 126.553). The total validation sample was randomly divided into Subsample A (n = 310) for EFA and Subsample B (n = 309) for CFA. Demographic and professional characteristics of the pretest and validation samples are presented in Table 1.

**Table 1:** Demographic and Professional Characteristics of the Pretest and Validation Samples

Variable	Pretest sample (*N* = 157)	Total sample (*N* = 619)	Subsample A (*N* = 310)	Subsample B (*N* = 309)
	*N* (%) /Mean (SD)			
Age	44.65(10.95)	42.29(11.10)	43.59(10.82)	42.00(11.39)
Sex				
Men	42(26.8)	134(21.6)	59(19)	75(24.3)
Women	115(73.2)	485(78.2)	251(81)	234(75.7)
Education				
High school or below (including high school)	0	30(4.8)	18(5.8)	12(3.9)
Junior college	10(6.37)	110(17.7)	49(15.8)	61(19.7)
Bachelor’s degree	106(67.52)	363(58.5)	179(57.5)	184(59.5)
Master’s degree	41(26.11)	111(17.9)	64(20.6)	47(15.2)
Doctoral degree (PhD)	0	5(0.8)	0	5(1.6)
Profession				
Physician	6(3.82)	43(7.0)	14(4.5)	29(9.4)
Nurse	48(30.57)	245(39.5)	124(40.0)	121(39.2)
Advanced practice nurse	13(8.28)	26(4.2)	12(3.9)	14(4.5)
HIV case manager	7(4.46)	10(1.6)	8(2.6)	2(0.6)
Other medical technologists	2(1.27)	25(4.0)	12(3.9)	7(2.3)
Pharmacist	77(49.04)	38(6.1)	20(6.5)	18(5.8)
Medical laboratory technologist	0(0)	27(4.4)	21(6.8)	6(1.9)
Therapist	0(0)	33(5.3)	13(4.2)	20(6.5)
Administrative staff	0(0)	105(17)	49(15.8)	56(18.1)
Intern	0(0)	5(0.8)	2(0.6)	3(1.0)
Care aide	0(0)	38(6.1)	17(5.5)	21(6.8)
Other	4(2.55)	24(3.9)	12(3.9)	12(3.9)
Infectious Disease Department				
Yes	26(16.56)	61(9.8)	34(11.0)	27(8.7)
No	131(83.44)	558(90.0)	276(89.0)	282(91.3)
Clinical Experience				
Less than 6 months	2(1.27)	52(8.4)	25(8.1)	27(8.7)
6 m-2y	5(3.18)	47(7.6)	24(7.7)	23(7.4)
2y-5y	10(6.37)	69(11.1)	28(9.0)	41(13.3)
5y-10y	29(18.47)	102(16.5)	50(16.1)	52(16.8)
10y-15y	31(19.75)	124(20.0)	63(20.3)	61(19.7)
15 + years	80(50.96)	225(36.3)	120(38.7)	105(34.0)
Participation in HIV-related training^1^				
Yes	106(67.5)	435(70.27)	228(73.5)	207(67.0)
No	51(32.5)	184(29.73)	82(26.5)	102(33.0)
Number of people with HIV served by the respondent in the past month	26.89 (104.88)	11.82 (126.553)	8.26(40.82)	15.40(174.47)
Number of people with HIV served by the respondent in the past week	19.63 (105.80)	3.28(32.942)	2.10(8.48)	4.47(45.85)
Total hours of participation in the HIV-related training courses in the past year	6.655 (13.98)	3.98(8.154)	4.74(10.61)	3.22(4.39)

^1^ Participation in HIV-related training: stigma and discrimination; infection control and universal precautions including post-exposure prophylaxis; informed consent, privacy, and confidentiality; stigma toward key populations

#### Item analysis (Pretest Sample, *n* = 157)

Item discrimination analysis conducted in the independent pretest sample showed that all items demonstrated statistically significant critical ratios except Item 6 (“If I discriminate against PWH, I will get into trouble or be punished”), which had a non-significant CR value of 1.003 and was removed. The remaining items demonstrated CR values ranging from − 2.635 to − 11.654 (*p* < .05 to *p* < .001). The overall Cronbach’s alpha for the retained items was 0.889, indicating good internal consistency. Detailed item analysis results are presented in Table 2.

**Table 2:** Item analysis of the WHO-based HIV stigma instrument (Pretest sample, *n* = 157)

Original item		Differentiation	Action 1	Congeniality	Action 2
English version	Mandarin version	Critical ratio		Cronbach’s α if item eliminated	
1–1. I feel worried when touching the clothing of people with HIV (PWH)	1.-1.觸碰到 HIV 感染者的衣服時, 讓我很擔心	-7.291***	Reserved	0.883	Reserved
1–2. I feel worried when dressing wounds of PWH	1.-2.為 HIV 感染者包紮傷口時, 讓我很擔心	-7.122***	Reserved	0.883	Reserved
1–3. I feel worried when drawing blood from PWH	1.-3.為 HIV 感染者抽血時, 讓我很擔心	-7.399***	Reserved	0.883	Reserved
1–4. I feel worried when measuring the temperature of PWH	1.-4.為 HIV 感染者量測體溫時, 讓我很擔心	-4.747***	Reserved	0.885	Reserved
2 − 1. I avoid physical contact when providing care to PWH	2.-1.在提供 HIV 感染者服務時, 會避免身體接觸	-11.654***	Reserved	0.880	Reserved
2–2. I wear an extra pair of gloves when providing care to PWH	2.-2.在提供 HIV 感染者服務時, 會多戴一層手套	-10.618***	Reserved	0.884	Reserved
2–3. I wear gloves for all procedures when interacting with PWH	2.-3.面對 HIV 感染者時, 會在進行任何服務時都戴手套	-9.124***	Reserved	0.884	Reserved
2–4. I apply different infection control measures to PWH compared to other patients	2.-4.會對 HIV 感染者使用和非感染者不同的感染管制措施	-10.223***	Reserved	0.883	Reserved
3 − 1. Healthcare workers are unwilling to provide services to PWH or those suspected of having HIV	3.-1.醫療照護相關人員不願意為HIV感染者或疑似感染者提供服務	-2.743**	Reserved	0.889	Reserved
3 − 2. Healthcare workers provide lower quality care to PWH or those suspected of having HIV compared to other patients	3.-2.醫療照護相關人員為HIV感染者或疑似感染者提供的服務比一般病人差	-2.652*	Reserved	0.889	Reserved
3–3. Healthcare workers speak negatively about PWH or those suspected of having HIV	3.-3.醫療照護相關人員說HIV感染者或疑似感染者的壞話	-2.635*	Reserved	0.890	Reserved
3–4. Healthcare workers hesitate to work with colleagues who are living with HIV	3.-4.醫療照護相關人員會猶豫跟感染HIV的同事一起工作	-5.192***	Reserved	0.887	Reserved
4 − 1. I worry that others will speak negatively about me because I provide care to PWH	4.-1.我擔心別人會因為我為HIV感染者提供服務而說我的壞話	-8.645***	Reserved	0.884	Reserved
4 − 2. I worry that my family or friends will avoid contact with me because I provide care to PWH	4.-2.我擔心家人或朋友會因為我為HIV感染者提供服務而避免和我接觸	-10.213***	Reserved	0.883	Reserved
4 − 3. I worry that my colleagues will avoid contact with me because I provide care to PWH	4.-3.我擔心同事會因為我為HIV感染者提供服務而避免和我接觸	-10.058***	Reserved	0.882	Reserved
5. My healthcare facility only conducts HIV testing with the patient’s informed consent.	5.我所在的醫療機構僅允許在病人知情的情況下, 為病人進行HIV檢測	-4.273***	Reserved	0.891	Reserved
6. If I discriminate against PWH, I would face consequences or punishment.	6.如果我歧視HIV感染者, 我會因此遇到麻煩或被處罰	1.003	Eliminated	-	-
7. My workplace provides sufficient protective equipment (e.g., gloves) to reduce the risk of HIV infection.	7.我所在的醫療單位有足夠的防護用品(例如, 手套)等, 以減少我被HIV感染的機率	-6.489***	Reserved	0.888	Reserved
8. My workplace has standard procedures or protocols to reduce the risk of HIV infection	8.我所在的醫療單位有標準的流程或規定, 以減少我被HIV感染的機率	-8.227***	Reserved	0.887	Reserved
9. My workplace has clear policies to protect PWH from discrimination	9.我所在的醫療單位有完善的規範準則, 以保護HIV感染者不受歧視	-6.633***	Reserved	0.889	Reserved
10. Most PWH do not care whether they transmit HIV to others	10.大多數HIV感染者並不在意自己是否會感染別人	-4.988***	Reserved	0.888	Reserved
11. PWH should feel ashamed of their behavior	11.HIV感染者應該為自己的行為感到羞恥	-6.468***	Reserved	0.886	Reserved
12. Most PWH have multiple sexual partners	12.大多數HIV感染者有許多性伴侶	-4.043***	Reserved	0.890	Reserved
13. People acquire HIV as a result of their own irresponsible behavior	13.病人感染HIV是他們自己不負責任的行為而造成的	-5.168***	Reserved	0.889	Reserved
14. HIV infection is a punishment for immoral behavior	14.感染HIV是對不良行為的一種懲罰	-7.549***	Reserved	0.885	Reserved
15. It is acceptable for women living with HIV to have children if they wish	15.如果HIV感染的女性有意願生小孩, 這樣的情形是可以的	-5.507***	Reserved	0.889	Reserved
16. If I had a choice, I would not provide care to people who inject drugs	16.如果我可選擇, 我不會為注射毒品者提供服務	-6.443***	Reserved	0.886	Reserved
17. If I had a choice, I would not provide care to men who have sex with men	17.如果我可選擇, 我不會為男男性行為者提供服務	-8.929***	Reserved	0.882	Reserved
18. If I had a choice, I would not provide care to sex workers	18.如果我可選擇, 我不會為性工作者提供服務	-9.946***	Reserved	0.882	Reserved
	Cronbach’s α			0.889	

* *p* < .05, ** *p* < .01, *** *p* < .001

#### Construct validity (Subsample A, *n* = 310)

##### Exploratory factor analysis

Sampling adequacy was supported by a Kaiser–Meyer–Olkin value of 0.849 and a significant Bartlett’s test of sphericity (χ² = 4887.093, df = 325, *p* < .001). An iterative item reduction process was performed. Item 15 was removed due to cross-loading and marginal primary loading below 0.50. Item 2 − 1 was subsequently removed because it loaded on a factor inconsistent with theoretical expectations.

The final EFA solution yielded a seven-factor structure explaining 72.98% of the total variance. The variance explained by each factor was 13.08%, 11.41%, 11.14%, 10.47%, 10.40%, 8.98%, and 7.50%, respectively. Standardized factor loadings ranged from 0.447 to 0.920. Most loadings exceeded 0.50; one item showed a loading slightly below 0.50 (0.447) but was retained due to theoretical relevance and absence of cross-loading.

The seven factors were interpreted as Fear, Extra Infection Precautions, Observed Enacted Stigma, Secondary Stigma, Health Facility Policies and Work Environment, Attitudes toward PWH, and Willingness to Treat Key Populations. The factor loadings, eigenvalues, and variance explained for the retained factors are presented in Table 3.

**Table 3:** Exploratory Factor Analysis of the Final Seven-Factor Structure (Subsample A, *n* = 310)

Item	Factor 6: Attitudes towards PWH	Factor 3: Observed enacted stigma	Factor 5: Health Facility Policies and Work Environment	Factor 4: Secondary stigma	Factor 1: Fear	Factor 2: Extra infection precautions	Factor 7: Willingness to treat key populations
1.-1.					0.698		
1.-2.					0.836		
1.-3.					0.759		
1.-4.					0.686		
2.-2.						0.702	
2.-3.						0.830	
2.-4.						0.769	
3.-1.		0.798					
3.-2.		0.861					
3.-3.		0.798					
3.-4.		0.790					
4.-1.				0.879			
4.-2.				0.903			
4.-3.				0.920			
5.			0.703				
7.			0.867				
8.			0.899				
9.			0.802				
10.	0.641						
11.	0.765						
12.	0.809						
13.	0.870						
14.	0.748						
16.							0.447
17.							0.823
18.							0.810
% of variance	13.079173	11.406431	11.141387	10.471230	10.399092	8.983053	7.498283
Eigenvalue	7.587	2.991	2.394	2.105	1.776	1.086	1.035
Cumulative %	13.079173	24.485604	35.626992	46.098222	56.497314	65.480367	72.978651
Kaiser-Mayer-Olkin measure of sampling adequacy (KMO)					0.849		
Bartlett’s test of sphericity					4887.093***		

Item 16 demonstrated a loading slightly below the conventional 0.50 threshold but was retained due to theoretical relevance and absence of problematic cross-loading

***: *p*<.001

##### Confirmatory factor analysis and convergent validity

The seven-factor correlated first-order model demonstrated acceptable fit: χ² = 682.579, df = 278, χ²/df = 2.455, CFI = 0.916, TLI = 0.902, GFI = 0.849, RMSEA = 0.069 (90% CI [0.062, 0.075]), and SRMR = 0.053. All standardized factor loadings were statistically significant (*p* < .001) and ranged from 0.513 to 0.980.

**Fig. 1:**
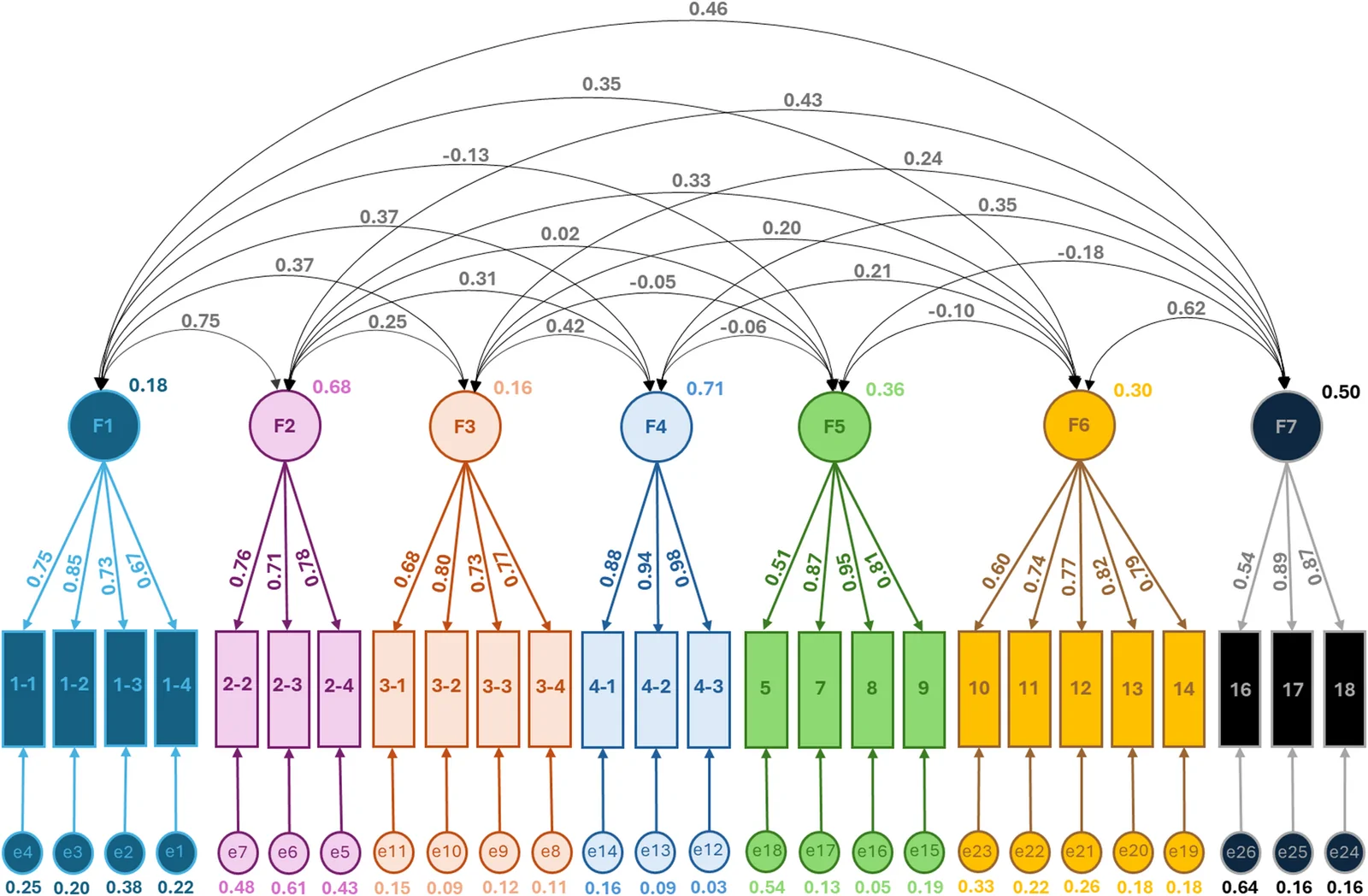
Standardized confirmatory factor analysis model of the seven-factor structure of the Traditional Chinese WHO/USAID Health Facility Staff HIV Stigma and Discrimination Questionnaire. Values represent standardized factor loadings and latent construct correlations. Abbreviations: F1 = Fear; F2 = Extra Infection Precautions; F3 = Observed Enacted Stigma; F4 = Secondary Stigma; F5 = Health Facility Policies and Work Environment; F6 = Attitudes toward PWH; F7 = Willingness to Treat Key Populations

Composite reliability values ranged from 0.794 to 0.954, exceeding the recommended threshold of 0.70. Average variance extracted (AVE) values ranged from 0.557 to 0.873, supporting convergent validity of all seven domains. Detailed CFA results, standardized factor loadings, CR, and AVE values are presented in Table 4. The standardized seven-factor measurement model is presented in Fig. 1.

**Table 4:** Confirmatory Factor Analysis Results and Convergent Validity of the Seven-Factor Model

WHO Domains	Item	Unstandardized Regression Weights	S.E.	C.*R*.	*P*	Standardized Regression Weights	SMC	CR	AVE
Fear	1.-1.	1.33	0.12	11.40	***	0.75	0.57	0.839	0.567
	1.-2.	1.68	0.14	12.42	***	0.85	0.72		
	1.-3.	1.52	0.14	11.05	***	0.73	0.53		
	1.-4.	1.00				0.67	0.46		
Extra Infection Precautions	2.-2.	0.97	0.08	12.33	***	0.76	0.57	0.794	0.563
	2.-3.	0.96	0.08	11.69	***	0.71	0.51		
	2.-4.	1.00				0.78	0.61		
Observed Enacted Stigma	3.-1.	0.91	0.08	11.36	***	0.68	0.47	0.834	0.557
	3.-2.	0.99	0.08	13.20	***	0.80	0.64		
	3.-3.	0.92	0.08	12.17	***	0.73	0.53		
	3.-4.	1.00				0.77	0.60		
Secondary Stigma	4.-1.	0.86	0.03	28.48	***	0.88	0.77	0.954	0.873
	4.-2.	0.99	0.03	37.59	***	0.94	0.89		
	4.-3.	1.00				0.98	0.96		
Health Facility Policies and Work Environment	5.	0.73	0.08	9.32	***	0.51	0.26	0.874	0.644
	7.	1.05	0.06	18.31	***	0.87	0.76		
	8.	1.19	0.06	19.88	***	0.95	0.91		
	9.	1.00				0.81	0.66		
Attitudes toward PWH	10.	0.79	0.07	10.56	***	0.60	0.36	0.863	0.559
	11.	0.95	0.07	13.39	***	0.74	0.55		
	12.	1.12	0.08	14.05	***	0.77	0.60		
	13.	1.11	0.07	15.01	***	0.82	0.67		
	14.	1.00				0.79	0.62		
Willingness to Treat Key Populations	16.	0.73	0.07	9.74	***	0.54	0.29	0.820	0.614
	17.	1.08	0.06	17.09	***	0.89	0.79		
	18.	1.00				0.87	0.76		

Standardized regression weights represent standardized factor loadings

*S.E*. standard error, *C.R.* critical ratio, *SMC* squared multiple correlation, *CR* composite reliability, *AVE* average variance extracted

CR values ≥ 0.70 indicate acceptable internal consistency, and AVE values ≥ 0.50 indicate adequate convergent validity

##### Discriminant validity

Discriminant validity was evaluated using the Fornell–Larcker criterion. As shown in Table 5, the square root of the AVE for each construct exceeded its correlations with all other constructs, supporting acceptable discriminant validity across the seven-factor model. The strongest association was observed between Fear and Extra Infection Precautions (*r* = .748); however, the square roots of their AVEs (0.753 and 0.750, respectively) remained slightly higher than the inter-construct correlation, indicating that the two constructs were empirically distinct despite their conceptual relatedness.

**Table 5:** Convergent and Discriminant Validity of the Seven-Factor Model

Domains	AVE	Fear	Extra Infection Precautions	Observed Enacted Stigma	Secondary Stigma	Health Facility Policies and Work Environment	Attitudes toward PWH	Willingness to Treat Key Populations
Fear	0.567	**0.753**						
Extra Infection Precautions	0.563	0.748	**0.750**					
Observed Enacted Stigma	0.557	0.365	0.251	**0.746**				
Secondary Stigma	0.873	0.371	0.310	0.421	**0.934**			
Health Facility Policies and Work Environment	0.644	-0.126	0.024	-0.048	-0.063	**0.802**		
Attitudes toward PWH	0.559	0.351	0.330	0.198	0.209	-0.099	**0.748**	
Willingness to Treat Key Populations	0.614	0.462	0.432	0.238	0.351	-0.183	0.619	**0.784**

Values on the diagonal (bold) represent the square root of AVE for each construct; off-diagonal values represent inter-construct correlation

*AVE* avarage variance extracted

##### Convergent validity with HPASS (external comparator)

Using the validation subsample (Subsample B, *n* = 309), conceptually aligned domains demonstrated moderate to strong correlations with HPASS constructs, supporting convergent validity. Attitudes toward PWH showed strong associations with stereotyping (*r* = .691, *p* < .01) and moderate associations with prejudice (*r* = .470, *p* < .01) and discrimination (*r* = .362, *p* < .01). Willingness to treat key populations was moderately to strongly correlated with prejudice (*r* = .532, *p* < .01) and discrimination (*r* = .520, *p* < .01).

Domains reflecting affective and behavioral components, including fear and extra infection precautions, showed moderate correlations with HPASS (r range = 0.260–0.548, all *p* < .01). In contrast, structural domains such as observed enacted stigma and health facility policies demonstrated weak or non-significant correlations (r range = − 0.001 to 0.218). Correlations between the WHO stigma domains and HPASS constructs are presented in Table 6.

**Table 6:** Correlations Between WHO Stigma Domains and HPASS Constructs (subsample B *N* = 309)

WHO Domains	HPASS–Prejudice	HPASS–Stereotyping	HPASS–Discrimination
Fear	0.548**	0.260**	0.365**
Extra Infection Precautions	0.467**	0.333**	0.324**
Observed Enacted Stigma	0.138*	0.080	0.099
Secondary Stigma	0.331**	0.152**	0.266**
Health Facility Policies and Work Environment	0.162**	− 0.001	0.218**
Attitudes toward PWH	0.470**	0.691**	0.362**
Willingness to Treat Key Populations	0.532**	0.367**	0.520**

* *p* < .05 ** *p* < .01 (two-tailed)

Overall, these findings support both convergent and discriminant validity, indicating that the WHO-based instrument captures both overlapping and distinct dimensions of stigma compared with HPASS.

#### Reliability

The final scale demonstrated good internal consistency, with Cronbach’s α = 0.889. Composite reliability values ranged from 0.794 to 0.954, indicating satisfactory to excellent internal consistency across constructs.

## Discussion

This study aimed to culturally adapt and psychometrically validate a Traditional Chinese version of the WHO/USAID Health Facility Staff HIV Stigma and Discrimination Questionnaire for use among healthcare workers in Taiwan [13]. The adapted instrument demonstrated strong psychometric performance in Taiwanese healthcare settings, with both exploratory and confirmatory factor analyses supporting a coherent seven‑factor structure. Discriminant validity was supported by the Fornell–Larcker criterion, indicating that the seven domains represent related but distinct dimensions of HIV-related stigma. Notably, Fear and Extra Infection Precautions showed the strongest association among the identified domains, which is theoretically expected because concerns regarding HIV transmission may influence precautionary behaviors in clinical practice. Nevertheless, these constructs remained empirically distinguishable, supporting the multidimensional structure proposed by the WHO stigma framework. These findings offer the first evidence that the WHO instrument can be reliably applied in Taiwan following systematic cultural adaptation. Beyond psychometric validation, the results advance the conceptualization of stigma measurement by empirically supporting a multidimensional, health facility–based model. The identified domains encompass fear of HIV transmission, unnecessary precautions, attitudes toward PWH and key populations, observed enacted stigma, and institutional policies, and they align with contemporary stigma frameworks that emphasize stigma as a multilevel phenomenon shaped by individual, interpersonal, and structural forces [5, 9]. This extends prior measurement approaches that focus primarily on individual attitudes, such as the HPASS, and reinforces that stigma in healthcare settings is shaped by interpersonal norms and institutional structures that cannot be captured by attitude‑based tools alone.

In Taiwan, several structural features of the healthcare system may shape how HIV-related stigma is produced and sustained within facilities. The centralized HIV care delivery model and mandatory case reporting system may heighten healthcare workers’ perceptions of occupational responsibility and legal accountability, which in turn can influence precautionary behaviors and concerns about confidentiality. Prior qualitative studies in Taiwan have documented that providers sometimes experience tension between maintaining patient privacy and fulfilling administrative requirements, suggesting that institutional procedures may inadvertently reinforce stigma-related practices [23]. Moreover, universal health coverage and high patient volumes in hospital-based HIV care may create workflow pressures that limit opportunities for stigma-reduction training or reflective practice. These contextual factors help explain why structural domains identified in the present instrument, including institutional policies, observed enacted stigma, and secondary stigma, emerged as distinct constructs and demonstrated weak correlations with individual-level attitudes measured by HPASS. Taken together, these findings highlight that stigma in Taiwanese healthcare settings is shaped not only by personal beliefs but also by organizational norms, administrative procedures, and system-level expectations.

Beyond these structural influences, a notable contribution of this study is the identification of observed enacted stigma as a distinct and measurable construct. Observed stigma captures discriminatory behaviors witnessed among colleagues and reflects the interpersonal and normative processes that operate within healthcare teams. Evidence from an Australian intervention study demonstrated that shifts in perceived peer norms independently predicted reductions in stigmatizing attitudes among healthcare workers [30], illustrating how normative expectations within healthcare teams can meaningfully shape provider behavior. Consistent with this perspective, studies from Egypt and China have shown that healthcare workers frequently observe discriminatory behaviors in clinical settings, which may normalize stigma and reinforce its persistence [31, 32]. In the present study, observed stigma showed weak correlations with HPASS constructs, indicating that it captures interpersonal and normative dynamics not reflected in traditional attitude‑based measures. This reinforces the importance of assessing social norms within healthcare teams when designing stigma‑reduction interventions.

This study also provides empirical support for institutional policies and work environment as components of structural stigma. Structural stigma refers to inequities embedded in organizational rules, policies, and procedures that shape provider behavior and patient experiences [33, 34]. Prior research has shown that institutional culture, infection‑control protocols, and workplace policies can either reinforce or mitigate stigmatizing practices [18, 19]. In this study, institutional domains emerged as an independent factor with limited overlap with individual‑level measures, underscoring the need to assess structural conditions separately when evaluating stigma in healthcare settings. This consideration is particularly relevant in Taiwan, where centralized HIV care delivery, mandatory reporting systems, and documented concerns about privacy and disclosure among PWH may influence perceptions of risk and institutional practices [21, 23].

The pattern of associations with HPASS further clarifies the complementary roles of these instruments. Domains reflecting individual attitudes, such as attitudes toward PWH and willingness to treat key populations, showed moderate to strong correlations with HPASS constructs, supporting convergent validity. In contrast, domains related to observed stigma and institutional policies demonstrated weak or negligible correlations, indicating that they capture distinct dimensions of stigma. These findings suggest that the WHO-based instrument should be viewed as complementary to, rather than a replacement for, HPASS. Together, the two instruments enable a more comprehensive assessment of stigma across individual, interpersonal, and structural levels.

From a practical standpoint, this validated instrument provides a valuable tool for assessing HIV-related stigma across multiple levels within healthcare settings. By capturing individual attitudes, interpersonal dynamics, and institutional conditions within a single framework, it enables healthcare facilities to identify specific drivers of stigma and target quality-improvement initiatives accordingly. Because the validation sample included both clinical and non-clinical personnel, users of the instrument should consider potential differences in occupational exposure, HIV-related training, and patient contact when interpreting scores across professional groups. Until measurement invariance has been formally established, comparisons between different healthcare occupations should be interpreted cautiously, as observed differences may reflect both variation in stigma-related attitudes and profession-specific workplace experiences. For example, hospitals may use the instrument to identify units with elevated fear‑based behaviors, inconsistent policy implementation, or gaps in infection‑control training. The inclusion of institutional domains is particularly important for identifying structural gaps, such as inconsistent policy implementation or inadequate infection‑control training, that may contribute to stigma.

At the policy level, the instrument offers a standardized approach for generating facility‑level stigma indicators that may have for incorporation into national monitoring systems following additional validation in geographically diverse and representative healthcare settings. Countries such as Thailand have successfully integrated adapted versions of the WHO tool into routine surveillance [15], demonstrating its feasibility for large‑scale implementation. In Taiwan, where centralized HIV care and reporting structures already exist, integrating this instrument into routine monitoring could support systematic tracking of stigma trends, evaluation of stigma‑reduction programs, and evidence‑based policy development.

This study has several strengths. It represents the first cultural adaptation and validation of the WHO/USAID health‑facility stigma instrument in Taiwan, addressing a critical measurement gap in the region. The use of both exploratory and confirmatory factor analyses provides rigorous evidence for the instrument’s multidimensional structure, while the inclusion of HPASS enables a robust assessment of convergent and discriminant validity. The large, multi-institutional sample enhances the diversity of participating healthcare workers and healthcare settings, and the systematic adaptation process followed established cross‑cultural guidelines [20].

Several limitations should also be considered. First, the use of convenience sampling may limit representativeness, particularly for smaller facilities or non‑clinical staff groups. Consequently, caution is warranted when considering the use of the instrument for benchmarking or large-scale monitoring purposes, and additional validation in representative healthcare samples is needed. Second, the validation sample included healthcare personnel from diverse professional backgrounds, including both clinical and non-clinical staff. Although this composition is consistent with the intended application of the WHO/USAID Health Facility Staff HIV Stigma and Discrimination Questionnaire at the health facility level, differences in occupational exposure, HIV-related training, and patient contact may influence stigma-related responses. Measurement invariance across professional groups was not formally examined in the present study. Third, the cross‑sectional design precludes assessment of test–retest reliability and limits conclusions about the temporal stability of the instrument. Fourth, all measures relied on self‑report and may be subject to social desirability bias, especially for sensitive items related to attitudes or observed stigma. Finally, although the cultural adaptation process was rigorous, additional qualitative work may be needed to further explore item interpretation across different healthcare professions and institutional contexts.

Future research should evaluate temporal stability, measurement invariance across professional groups, and the instrument’s responsiveness to stigma-reduction interventions. Studies conducted in a broader range of healthcare settings and linked with patient-reported experiences and clinical outcomes may further strengthen evidence for the instrument’s applicability and practical value.

## Conclusions

In conclusion, this study demonstrates that HIV‑related stigma in healthcare settings is best understood as a multilevel phenomenon shaped by the interaction of individual beliefs, social norms, and institutional structures. The validated Traditional Chinese version of the WHO/USAID Health Facility Staff HIV Stigma and Discrimination Questionnaire provides a comprehensive and contextually appropriate tool for measuring these dimensions in Taiwan. By enabling systematic assessment of individual, interpersonal, and structural drivers of stigma, the instrument offers a foundation for strengthening routine monitoring, guiding targeted stigma‑reduction strategies, and informing evidence‑based policy development. Advancing stigma measurement at the health‑facility level is essential for improving the quality and equity of HIV care, and future efforts should integrate these metrics into research, clinical practice, and national health system planning to ensure sustained progress toward stigma‑free healthcare environments.

## Supplementary Information

Supplementary Material 1.

## Data Availability

To protect participant anonymity, the full datasets generated and analyzed in this study are not publicly available, but they may be obtained from the corresponding author upon reasonable request.

## Abbreviations

PWH: People With HIV
ECDC: European Centre for Disease Prevention and Control
HPASS: Health Care Provider HIV/AIDS Stigma Scale
TW-HPASS: Traditional Chinese version of HPASS
USAID: United States Agency for International Development
CVI: Content Validity Index
EFA: Exploratory Factor Analysis
CFA: Confirmatory Factor Analysis
CR: Composite Reliability
AVE: Average Variance Extracted
I-CVI: Item-level Content Validity Indices
