# Supplementary material for: Psychometric validation of a multidimensional health facility HIV stigma instrument among healthcare workers in Taiwan
Source: BMC Public Health. 2026 Jun 27;26:2826. doi: 10.1186/s12889-026-28284-2 (PMC13644188; doi:10.1186/s12889-026-28284-2)
Supplement: Supplementary file 1 [file 12889_2026_28284_MOESM1_ESM.docx]

**Supplementary**

**Table S1.** **Table S1. Correlations Between WHO Stigma Domains and HPASS Constructs (N = 619)**

| Domains | **1** | **2** | **3** | **4** | **5** | **6** | **7** | **8** | **9** | **10** |
| --- | --- | --- | --- | --- | --- | --- | --- | --- | --- | --- |
| 1. Prejudice | — |  |  |  |  |  |  |  |  |  |
| 2. Stereotyping | .564** | — |  |  |  |  |  |  |  |  |
| 3. Discrimination | .622** | .491** | — |  |  |  |  |  |  |  |
| 4. Fear | .571** | .303** | .404** | — |  |  |  |  |  |  |
| 5. Extra Infection Precautions | .507** | .367** | .371** | .565** | — |  |  |  |  |  |
| 6. Observed Enacted Stigma | .263** | .202** | .209** | .372** | .240** | — |  |  |  |  |
| 7. Secondary Stigma | .350** | .188** | .281** | .345** | .251** | .698** | — |  |  |  |
| 8. Health Facility Policies | -.187** | .020 | -.170** | -.135* | .020 | -.102* | -.123** | — |  |  |
| 9. Attitudes toward PWH | .467** | .703** | .408** | .313** | .338** | .280** | .240** | -.080* | — |  |
| 10. Willingness to Treat Key Populations | .585** | .430** | .518** | .431** | .396** | .303** | .346** | -.193** | .538** | — |

* p < .05	** p < .01

**Table S2. Exploratory Factor Analysis and Item Reduction Process**

| **Stage** | **KMO** | **Bartlett’s χ²** | **df** | **p** | **No. of Factors** | **Item Removed** | **Criteria for Removal** |
| --- | --- | --- | --- | --- | --- | --- | --- |
| Item Analysis | – | – | – | – | – | Item 6 | Low item discrimination |
| Iteration 1 (Initial EFA) | .857 | 5174.609 | 378 | < .001 | 7 | Item 15 | Cross-loading with marginal primary loading (< .50) and limited conceptual coherence with the core latent construct |
| Iteration 2 | .859 | 5086.423 | 351 | < .001 | 7 | Item 2-1 | Loaded primarily on a factor inconsistent with the a priori theoretical construct, indicating construct misclassification |
| Final Model | .849 | 4887.093 | 325 | < .001 | 7 | None | Final Model  All retained items demonstrated primary loadings ≥ .50 except Item 16 (.447), which was retained based on theoretical relevance and absence of problematic cross-loading. |
